# Cortical vein thrombosis in adult patients of cerebral venous sinus thrombosis correlates with poor outcome and brain lesions: a retrospective study

**DOI:** 10.1186/s12883-017-0995-y

**Published:** 2017-12-15

**Authors:** Jiahui Liang, Hongbing Chen, Zhuhao Li, Shaofu He, Boning Luo, Shujin Tang, Wenjin Shang, Jinsheng Zeng

**Affiliations:** Department of Neurology and Stroke Center, The First Affiliated Hospital, Sun Yat-Sen University, No. 58 Zhongshan Road II, Guangzhou, 510080 People’s Republic of China

**Keywords:** Cerebral venous sinus, Thrombosis, Cortical vein, Hemorrhage, Infarction, Adult, Outcome

## Abstract

**Background:**

Cortical vein thrombosis (CVT) receives little attention in adult patients with cerebral venous sinus thrombosis (CVST). This study aimed to investigate the clinical and radiological features of adult CVST patients with concomitant CVT.

**Methods:**

From May 2009 to May 2016, we recruited 44 adult CVST patients (diagnosed within 1 month of onset; 33.8 ± 14.0 years of age, 28 males). CVT was primarily confirmed using computed tomography venography and magnetic resonance imaging sequence of contrast enhanced three dimensions magnetization prepared rapid acquisition with gradient echo. Patients with concomitant CVT were divided into the CVT group; otherwise, the patients were placed into the non-CVT group. The clinico-radiological characteristics were compared between the two groups.

**Results:**

The CVT group included 27 patients (61.4%), and the non-CVT group included 17 patients (38.6%). Seizure (63.0% versus 11.8%), focal neurological deficits (44.4% versus 5.9%), and consciousness disorders (33.3% versus 0) occurred more frequently in the patients in the CVT group than in those of the non-CVT group (*P* < 0.05). The modified Rankin Scale (mRS) score at discharge was higher for the CVT group patients (median 2, range 1–4) than for the non-CVT group patients (median 0, range 0–4) (*P* < 0.001). Venous infarction (63.0% versus 11.8%), parenchymal hemorrhage (40.7% versus 5.9%), and subarachnoid hemorrhage (22.2% versus 0) were identified more frequently in the CVT group than in the non-CVT group (*P* < 0.05).

**Conclusions:**

This study demonstrates that concomitant CVT is a common finding in adult patients with CVST and is associated with severe clinical manifestations, poor short-term outcomes, and brain lesions.

**Electronic supplementary material:**

The online version of this article (10.1186/s12883-017-0995-y) contains supplementary material, which is available to authorized users.

## Background

Thrombosis of the cerebral venous system is an important cerebrovascular disease [1] that can lead to severe brain injuries (such as venous infarction and parenchymal hemorrhage) and disability. Cerebral venous thrombosis has been reported to usually involve the venous sinuses [2, 3]. Only a few cases with isolated cortical venous thrombosis (ICVT) in which the major sinuses are not involved have been reported [4, 5]. The International Study on Cerebral Vein and Dural Sinus Thrombosis (ISCVT), a large prospective multi-central observational study, showed that 17.1% of patients with cerebral venous sinus thrombosis (CVST) had concomitant cortical venous thrombosis (CVT) accompanied by occlusion of the major sinuses [3]. In our study, we defined CVT as cortical venous thrombosis with concomitant occlusion of the major sinuses and ICVT as isolated cortical venous thrombosis without occlusion of the major sinuses. The diagnosis of CVT is challenging due to the relatively smaller sizes of the cortical veins compared with that of the cerebral venous sinuses. In early studies, CVT was usually identified using percutaneous puncture cerebral angiography [1], which was not a conventional vascular assessment method. Thus, we speculated that the incidence of concomitant CVT in CVST patients might have been underestimated in the past. With the development of non-invasive neuroimaging techniques (e.g., magnetic resonance imaging [MRI] and computed tomography [CT]) and improved recognition of CVT, a greater incidence of CVT has been diagnosed with contemporary neuroimaging techniques compared to the past decade. In a recent study that included pediatric CVST patients [6], concomitant CVT was found to be not rare (24%) and was associated with seizures and brain lesions. However, the significance of concomitant CVT in adult CVST patients is not clear. Thus, we used contemporary neuroimaging techniques to identify CVT, to investigate the associations of concomitant CVT with the clinico-radiological characteristics of adult CVST patients, and to further explore the pathophysiological mechanisms of cerebral venous thrombosis in adult patients.

## Methods

### Patients

According to the digital medical records, 51 adult patients (>18 years old) with CVST were admitted to the Department of Neurology and Stroke Center of the First Affiliated Hospital of Sun Yat-Sen University between May 2009 and May 2016. Patients were included if they met the following criteria: 1) CVST was diagnosed within 1 month of symptom onset; 2) the patient underwent cranial non-enhanced CT and conventional MRI; and 3) the patient underwent cranial computed tomography venography (CTV) or contrast-enhanced magnetic resonance venography (CE-MRV) and contrast-enhanced three dimensional magnetic prepared rapid acquisition gradient echo imaging (CE-3D-MPRAGE). We excluded patients with the following criteria: 1) insufficient clinico-radiological assessments; 2) missing source neuroimaging data; or 3) poor imaging data quality. This study was approved by the ethics committee of the First Affiliated Hospital of Sun Yat-Sen University. Because this study was retrospectively designed, informed consent was not required.

### Neuroimaging assessments

MRI was performed using a 3.0-T magnetic resonance scanner (MAGNETOM Trio Tim, Siemens Healthcare, Erlangen, Germany). All patients underwent conventional T1-weighted (T1WI), T2-weighted (T2WI) and fluid-attenuated inversion recovery (FLAIR) cranial MRI. Most patients underwent CE-MRV and CE-3D-MPRAGE. A 64-slice spiral CT scanner (Aquilion64, Toshiba Medical Systems, Tokyo, Japan) was used to perform the non-enhanced cranial CT in all patients and the CTV in some patients (see Table 1 and Additional file 1).

**Table 1:** Clinical characteristics

	Total	CVT group	Non-CVT group	*P* Value
	(*n* = 44)	(*n* = 27)	(*n* = 17)	
Age (mean ± SD), y	33.8 ± 14.0	32.6 ± 13.5	35.7 ± 14.9	0.484
Male, *n* (%)	28(63.6)	19(70.4)	9(52.9)	0.242
Duration from onset of symptoms to hospital arrival (median, range)	9,1~30	7,1~30	14,4~30	0.116
Clinical symptoms, *n* (%)				
Headache	39(88.6)	22(81.5)	17(100)	0.139
Seizure	19(43.2)	17(63.0)	2(11.8)	0.001
Focal neurological deficit	13(29.5)	12(44.4)	1(5.9)	0.006
Consciousness disorders	9(20.5)	9(33.3)	0(0)	0.008
Etiology, *n* (%)				0.493
Infection	5(11.4)	4(14.8)	1(5.9)	
Pregnancy/puerperium	5(11.4)	3(11.1)	2(11.8)	
Coagulation dysfunction	16(36.4)	11(40.7)	5(29.4)	
Autoimmune diseases	5(11.4)	3(11.1)	2(11.8)	
Malignancy	4(9.1)	3(11.1)	1(5.9)	
Undetermined source	9(20.5)	3(11.1)	6(35.3)	
Treatment, *n* (%)				
Anticoagulation	34(77.3)	21(77.8)	13(76.5)	1.000
Decompression Surgery	1(3.7)	1(3.7)	0(0)	1.000
mRS at discharge (median, range)	1,0~6	2,1~4	0,0~4	
0~2, *n* (%)	31(70.5)	16(59.3)	15(88.2)	0.040
3~6, *n* (%)	13(29.5)	11(40.7)	2(11.8)	

CVT indicates cortical vein thrombosis; *mRS* modified Rankin Scale, *SD* standard deviation

A senior neuroradiological doctor (B.L.) diagnosed CVST according to the previously described criteria of detection of a cord-like filling defect (a differential diagnosis from the nodule-like filling defects of arachnoid granulations) in a cerebral venous sinus site (i.e., the superior sagittal sinus, transverse sinuses, sigmoid sinuses, or the straight sinus) on CE-MRV or CTV with or without a concomitant cord-like hyperintensity on conventional MRI or a cord-like hyperdensity on non-enhanced CT in one of the cerebral venous sinus sites [1, 7]. The patients were divided into the CVT group if they had concomitant CVT or into the non-CVT group if no CVT was detected.

**Fig. 1:**
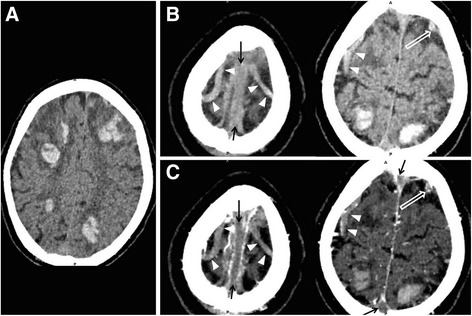
Typical images of the concomitant cortical vein thrombosis on CT and CT venography in a 61-year-old male patient with cerebral venous sinus thrombosis. Non-enhanced CT reveals multiple hemorrhages in bilateral frontal and parietal lobes (**a**). Cord-like (**b**, white arrowheads) and dot-like (**b**, hollow white arrow) hyperdensity (representing acute thrombosis) in the sites of cortical veins can be observed in non-enhanced CT. Non-enhanced CT also reveals the cord-like hyperdensity in the site of superior sagittal sinus (**b**, black arrows). On the source images of CT venography (**c**), filling defects of contrast agent can be detected in the sites of cortical veins (white arrowheads and hollow white arrow) and superior sagittal sinus (black arrows)

**Fig. 2:**
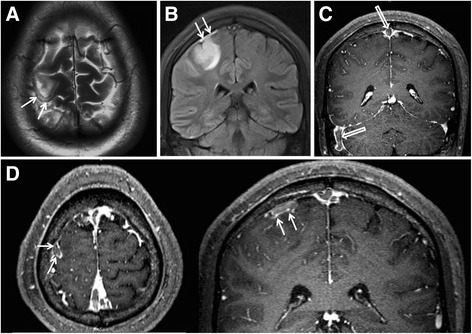
Typical images of the concomitant cortical vein thrombosis on conventional magnetic resonance imaging and contrast enhanced three dimensional-magnetization prepared rapid acquisition with gradient echo (CE-3D-MPRAGE) in a 23-year-old male patient with cerebral venous sinus thrombosis. The images of T2-weighted imaging (T2WI, **a**) and fluid-attenuated inversion recovery (FLAIR, **b**) show a cord-like hyperintensity (white arrows, representing a sub-acute thrombosis) of a right cortical vein. Venous infarction can be observed on FLAIR (**b**). The thin-thickness images of multiplanner reconstruction based on CE-3D-MPRAGE source images reveal the filling defects of contrast agent in the superior sagittal sinus and the right sigmoid sinus (**c**, hollow white arrows) and in the site of the aforementioned cortical vein (**d**, white arrows) presenting with a cord-like hyperintensity on T2WI and FLAIR

A senior cerebrovascular doctor (H.C.) and another senior neuroradiological doctor (Z.L.) who were blinded to the clinical data independently analyzed the imaging data to diagnose CVT according to the following criteria (Figs. 1 and 2): 1) a cord- or dot-like hyperintensity was observed on conventional MRI in a major cortical vein site; [8] 2) non-enhanced CT revealed a cord-like or dot-like hyperdensity [9] (65–72 Hounsfield units) [10] that was significantly higher than the major intracranial arteries in the major cortical vein site; or 3) the source images or multiplanar reconstructed (MPR) CTV or CE-3D-MPRAGE images revealed filling defects in the superficial cortical veins [5, 11]. CVT was diagnosed if the image data matched the third criterion and the first or the second criteria. If a discrepancy existed between two doctors’ findings, the final decision was made after a consensus was reached.

According to the neuroimaging results, we extracted information about intracranial abnormalities that were secondary to the cerebral venous thrombosis, which included brain lesions (venous infarctions and parenchymal hemorrhages), subarachnoid hemorrhages, and subdural hemorrhages. To differentiate baseline edema and infarction on the MRI, the acute infarction was contrast-enhanced on the CTV or CE-3D-MPRAGE. Additionally, thrombi of the deep venous system were not scored as CVT.

### Clinical assessments

The patients received systematic laboratory tests that included complete blood counts, urinalysis, prothrombin time, activated partial prothrombin time, thrombin time, d-dimer, liver and kidney functions, anti-thrombin III, protein S, protein C, and indicators of vasculitis (i.e., erythrocyte sedimentation rate, high sensitivity C-reactive protein levels, antinuclear antibody levels, and anticardiolipin antibody levels). Other assessments, including 12-lead electrocardiography, chest X-ray, and transthoracic echocardiogram, were available for all patients.

From the medical records, we also extracted clinical information including gender, age, symptoms, duration from the onset of symptoms to hospital arrival and the modified Rankin Scale (mRS) score at discharge. The short-term outcome was defined using the mRS at discharge (0–2 for good and 3–6 for poor).

The risk factors of the cerebral venous thrombi were assigned into one of the following categories: infection; autoimmune disease; pregnancy/puerperium; coagulation dysfunction; malignancy; and undetermined source.

### Statistical analysis

All data were analyzed using the IBM SPSS software for Windows (version 23.0; SPSS Inc., Chicago, IL, USA). Continuous variables were summarized as the means ± standard deviations if they were normally distributed. Ranked data were expressed as the medians and ranges. The differences between groups were analyzed using the independent samples *t* test for continuous variables, the Mann-Whitney *U* test for ranked variables, and Fisher’s exact test or the Chi-square test for categorical variables. *P* values less than 0.05 (2-tailed) were considered significant.

## Results

According to the aforementioned inclusion and exclusion criteria, 44 CVST patients (age: 33.8 ± 14.0 years, range from 19 to 62 years; 28 males) were enrolled into this study. The median duration from the onset of symptoms to hospital arrival was 9 days (range from 1 to 30 days). The CVT group included 27 patients (61.4%), and the non-CVT group included 17 patients (38.6%) (Table 1). Seven patients were excluded due to insufficient radiological assessments (neither CTV nor CE-3D-MPRAGE was performed).

### Clinical characteristics

For all patients, headache (88.6%) and seizure (43.2%) were the two leading symptoms, followed by focal neurological deficits (29.5%) and consciousness disorders (20.5%). A definite risk factor for cerebral venous thrombosis could be determined in most patients (79.5%), including coagulation dysfunction (36.4%), infection (11.4%), pregnancy/puerperium (11.4%), autoimmune diseases (11.4%), and malignancy (9.1%), but no factor was determined in 20.5% of the patients despite complete assessments. Table 1 compares the clinical characteristics between the two patient groups. Seizure (63.0% versus 11.8%), focal neurological deficit (44.4% versus 5.9%), and consciousness disorders (33.3% versus 0) were identified more frequently in the patients in the CVT group than in the non-CVT group (*P* < 0.05). The mRS scores were higher for the CVT group patients at discharge (median 2, range 1–4) than for the non-CVT group patients (median 0, range 0–4) (*P* = 0.016). Patients with poor short-term outcomes were more common in the CVT group than in the non-CVT group (*P* = 0.040). Two patients in the CVT group (7.4%) died in the hospital, whereas all of the patients in the non-CVT group were discharged from our hospital alive. No other significant differences in clinical characteristics were found between the two groups of patients (*P* > 0.05).

### Radiological characteristics

The most common CVST site was the superior sagittal sinus (72.7%), followed by the left transverse sinus (43.2%), left sigmoid sinus (43.2%), right transverse sinus (36.4%), right sigmoid sinus (36.4%), and straight sinus (9.1%). Thus, only a small proportion (9.1%) of the included patients had a concomitant thrombus of the deep venous systems. Approximately half of the patients (47.7%) had brain lesions (venous infarction: 43.2% and cerebral hemorrhage: 27.3%). The brain lesions most commonly involved the frontal lobe (31.8%) and the parietal lobe (36.4%). Other intracranial abnormalities that were secondary to venous thrombosis were subarachnoid hemorrhages (13.6%) and subdural hemorrhages (4.5%). The radiological characteristics of the patients in the two groups are compared in Table 2. In total, 22 patients in the CVT group were diagnosed with CVT by CE-3D-MPRAGE, and the remaining patients were diagnosed by CTV. Additionally, the thrombosis involved the superior sagittal sinus (85.2% versus 52.9%, *P* = 0.047) and the right transverse sinus (48.1% versus 17.6%, *P* = 0.041) more frequently in the CVT group than in the non-CVT group. Venous infarction (63.0% versus 11.8%, *P* = 0.001) and parenchymal hemorrhage (40.7% versus 5.9%, *P* = 0.029) were identified more frequently in the CVT group patients than in the non-CVT group patients. The brain lesions mainly occurred in the frontal (51.9%) and parietal lobes (51.9%) in the patients in the CVT group, whereas no patients in the non-CVT group had brain lesions in the frontal or parietal lobes (*P* < 0.05). No other differences in the radiological characteristics were found between the two groups.

**Table 2:** Radiological characteristics

	Total	CVT group	Non-CVT group	*P* Value
	(*n* = 44)	(*n* = 27)	(*n* = 17)	
Brain lesions, *n* (%)	21(47.7)	19(70.4)	2(11.8)	< 0.001
Venous infarction	19(43.2)	17(63.0)	2(11.8)	0.001
Parenchymal hemorrhage	12(27.3)	11(40.7)	1(5.9)	0.029
Subarachnoid hemorrhage	6(13.6)	6(22.2)	0(0)	0.101
Subdural hemorrhage	2(4.5)	1(3.7)	1(5.9)	1.000
Location of brain lesions, *n* (%)				
Frontal lobe	14(31.8)	14(51.9)	0(0)	< 0.001
Parietal lobe	16(36.4)	16(59.3)	0(0)	< 0.001
Temporal lobe	5(11.4)	5(18.5)	0(0)	0.162
Occipital lobe	3(6.8)	1(1.8)	2(11.8)	0.675
Location of CVST, *n* (%)				
Superior sagittal sinus	32(72.7)	23(85.2)	9(52.9)	< 0.001
Left transverse sinus	19(43.2)	12(44.4)	7(41.2)	0.831
Right transverse sinus	16(36.4)	13(48.1)	3(17.6)	0.041
Left sigmoid sinus	19(43.2)	13(48.1)	6(35.3)	0.402
Right sigmoid sinus	16(36.4)	9(33.3)	7(41.2)	0.598
Straight sinus	4(9.1)	2(7.4)	2(11.8)	1.000
Total numbers of CVST (median, range)	2,1~5	3,1~4	2,1~3	0.166

CVT indicates cortical vein thrombosis; and *CVST* cerebral venous sinus thrombosis

## Discussion

In our study, a higher incidence (61.4%) of concomitant CVT was found among adult CVST patients than pediatric CVST patients (24%) and the rate reported in a previous study (17%) [3, 6], which might be due to the integration of multiple imaging techniques for the detection of CVTs in our study (see Additional file 1: Tables S1 and S2).

In a recent study of pediatric CVST patients [6], the frequencies of consciousness disorder (58% vs. 21%) and seizure (58% vs. 21%) rather than focal neurological deficits (41% vs. 31%) were significantly higher among patients with concomitant CVT than patients without concomitant CVT. Similarly, seizure and focal neurological deficits were common (67% and 62%, respectively) among isolated CVT patients according to a systemic review [4]. However, in contrast to this result for pediatric CVST patients with concomitant CVT, consciousness disorders are rare among isolated CVT patients (2%) [4]. Seizure and focal neurological deficits were also common among the adult CVST patients with concomitant CVT in our study (44.4% and 63%, respectively). These results were similar to the results from the pediatric CVST patients and isolated CVT patients, but the rate of consciousness disorder (33.3%) was lower than the rate among the pediatric CVST patients. Moreover, in our study, focal neurological deficit, seizure, and consciousness disorder were more common among the adult CVST patients with concomitant CVT than among those without concomitant CVT. In summary, concomitant CVT was associated with severe clinical manifestations in both pediatric and adult CVST patients, which suggested a similar pathophysiological mechanism. In our adult patients, concomitant CVT was also related to a poor short-term outcome.

Parenchymal hemorrhage and venous infarction (50% and 75%, respectively) are common findings among pediatric CVST patients with concomitant CVT [6]. Similarly, our study also demonstrated a high frequency of brain lesions (venous infarction: 63% and parenchymal hemorrhage: 40.7%) among adult CVST patients with concomitant CVT. In addition to the analogous clinical manifestations of pediatric and adult CVST patients with concomitant CVT, the similarities of both conditions in terms of radiological profiles suggest the same pathophysiological mechanisms. We speculated that the mechanisms were as follows. (1) The blood flows of the cortex and its adjacent white matter are drained to cortical veins via the superficial medullary veins, which have poor collaterals [12]. Blockage of the outlets of the superficial medullary veins by thrombi in the cortical veins results in severe congestion of the brain tissues within the drainage areas of the cortical veins, leading to venous infarction or parenchymal hemorrhage. (2) In the condition of CVST without concomitant CVT, thrombosis in the venous sinus that blocks the outlets of the cortical veins may not cause severe congestion due to the good collaterals between the cortical veins [13]. Therefore, in practice, we should focus on the early diagnosis and treatment of CVST to prevent thrombi from extending from the cerebral venous sinuses to the cortical veins and to reduce the risk of venous infarction or hemorrhage and improve the prognosis.

In our study, we have compared the frequency of thrombosis in each venous sinus between CVT patients and non-CVT patients. We discovered that the superior sagittal sinus was more frequently involved in the CVT patients than in the non-CVT patients. However, the frequency of thrombosis in each venous sinus does not indicate the severity of venous sinus thrombosis; it denotes the spatial distribution of venous sinus thrombosis. When evaluating the severity of venous sinus thrombosis, we compared the total number of cerebral venous sinuses involved in thrombosis, and we did not find a significant difference between the CVT patients and non-CVT patients. Furthermore, the duration from onset of symptoms to hospital arrival is not significantly different between CVT group and non-CVT group. Thus, it seems that the severity of the involvement of sinuses and the duration from onset of symptoms to hospital arrival do not contribute to CVT. Further studies are needed to explore the factors predicting CVT in CVST patients.

Anticoagulation is a cornerstone for the treatment of thrombosis of the cerebral venous system [1]. Other treatments include endovascular recanalization and surgical decompression [1, 14, 15]. Our results suggest that different pathophysiological mechanisms are operating in CVST patients with and without concomitant CVT. However, with regard to therapeutic strategies, the significance of these differences remains unclear and should be investigated in future studies. Additionally, the CVST patients with concomitant CVT in our study exhibited poor short-term outcomes at discharge, and the majority were administered anticoagulation therapy. Anticoagulation therapy alone may not be sufficient for these patients, and other treatments should be developed. A case series study that included 6 CVST patients with concomitant CVT and severe clinical manifestations demonstrated that combined intra-arterial and intravenous thrombolysis could markedly improve the outcomes (i.e., the mRS scores at 3 months were zero) [16]. Thus, this therapeutic strategy seems promising, but future prospective cohort studies with large sample sizes are needed to verify its safety and efficiency.

This study had some limitations. First, the study was a retrospective research study with a relatively small sample size. Our findings need to be verified by future prospective studies with large sample sizes. Second, the methods used to detect CVT were not entirely uniform. CE-3D-MPRAGE was performed in the majority of our patients and exhibited high efficacy for the detection of CVT in this study. T2^*^ gradient echo (T2* GE) imaging and susceptibility-weighted imaging (SWI) utilize the magnetic susceptibility effect (MSE) of hemoglobin products to detect thrombi [17]. A previous study [18] demonstrated that T2* GE and SWI were more capable of detecting ICVT in the early and late phases than standard MRI protocols. In contrast, MSEs of the skull and intracranial hemorrhages also present as hypotensive, which may lead to confusion in the identification of CVT if the thrombus is adjacent to the skull or to a hemorrhagic lesion. Additionally, among the CVT patients with concomitant occlusions of the major sinuses, we found a high rate (40.7%) of intracranial hemorrhaging. Unfortunately, not all of the included patients underwent T2* or SWI in their MRI protocols. Thirdly, the ICVT patients were not included in this study. Thus, we cannot compare with CVST patients. Moreover, future studies should be conducted to compare the diagnostic efficacies of CE-3D-MPRAGE and T2^*^GE or SWI for CVTs; the latter method is recommended for CVST patients according to a recent guideline [7]. Third, because our hospital is a regional tertiary referral center, we risk selection bias in which the severity and the incidence of concomitant cortical vein thrombus may be overestimated.

## Conclusions

In conclusion, a concomitant CVT was a common finding in adult CVST patients and was associated with severe clinical manifestations, a poor short-term outcome, and brain lesions. The differences in the clinico-radiological features of adult CVST patients with and without a concomitant CVT are suggestive of different pathophysiological mechanisms. Future studies are needed to explore the significance of our findings for the treatment and prognosis of adult patients with thrombosis of the cerebral venous system.

## Additional file

Additional file 1: Table S1. The use of CTV, MRV, and CE-3D-MPRAGE in two groups. Table S1 showed that the rates of CTV, MRV, and CE-3D-MPRAGE utilization in CVT group were 29.6%, 85.2% and 85.2% respectively, while the rates in non-CVT group were 23.5%, 100% and 100% respectively. **Table S2.** Positive rate of CVT of different neuroimaging tests in the patients of CVT group respectively. Table S2 demonstrated that positive rate of CTV was 75.0% and lower than CE-3D-MPRAGE (95.7%). (DOCX 16 kb)

## Abbreviations

CE-3D-MPRAGE: Contrast enhanced three dimensions magnetization prepared rapid acquisition with gradient echo
CE-MRV: Contrast enhanced magnetic resonance venography
CTV: computed tomography venography
CVST: Cerebral venous sinus thrombosis
CVT: Cortical venous thrombosis
FLAIR: Fluid-attenuated inversion recovery
ICVT: Isolated cortical venous thrombosis
ISCVT: The international study on cerebral vein and dural sinus Thrombosis
MRI: Magnetic resonance imaging
mRS: Modified Rankin Scale
MSE: Magnetic susceptibility effect
SWI: Susceptibility weighted imaging
T1WI: T1-weighted imaging
T2* GE: T2* gradient echo
T2WI: T2-weighted imaging
