# Supplementary material for: Cortical vein thrombosis in adult patients of cerebral venous sinus thrombosis correlates with poor outcome and brain lesions: a retrospective study
Source: BMC Neurol. 2017 Dec 15;17:219. doi: 10.1186/s12883-017-0995-y (PMC5732418; doi:10.1186/s12883-017-0995-y)
Supplement: Additional file 1: Table S1. — The use of CTV, MRV, and CE-3D-MPRAGE in two groups. Table S1 showed that the rates of CTV, MRV, and CE-3D-MPRAGE utilization in CVT group were 29.6%, 85.2% and 85.2% respectively, while the rates in non-CVT group were 23.5%, 100% and 100% respectively. Table S2. Positive rate of CVT of different neuroimaging tests in the patients of CVT group respectively. Table S2 demonstrated that positive rate of CTV was 75.0% and lower than CE-3D-MPRAGE (95.7%). (DOCX 16 kb) [file 12883_2017_995_MOESM1_ESM.docx]

**Additional File 1**

**Cortical Vein Thrombosis in Adult Patients of Cerebral Venous Sinus Thrombosis Correlates with Poor Outcome and Brain Lesions: a retrospective study. - - Additional File 1**

**Table S1. CTV, MRV, and CE-3D-MPRAGE in two groups**

|  | CVT group  (n=27) | Non-CVT group  (n=17) | *P* Value |
| --- | --- | --- | --- |
| CTV, n (%) | 8(29.6) | 4(23.5) | 0.924 |
| CE-MRV, n (%) | 23(85.2) | 17(100) | 0.260 |
| CE-3D-MPRAGE, n (%) | 23(85.2) | 17(100) | 0.260 |

CE-3D-MPRAGE indicates contrast enhanced three dimensional magnetization prepared rapid acquisition with gradient echo; CTV, computed tomography venography; CVT, cortical vein thrombosis; and CE-MRV, contrast enhanced magnetic venography.

**Table S2. Positive rate of CVT of different neuroimaging tests in the patients of CVT group**

|  | Positive rate of CVT, n (%) |
| --- | --- |
| CTV (n=8) | 6(75.0) |
| CE-3D-MPRAGE (n=23) | 22(95.7) |

CE-3D-MPRAGE indicates contrast enhanced three dimensional magnetization prepared rapid acquisition with gradient echo; CTV, computed tomography venography; and CVT, cortical vein thrombosis.
